# Development of family‐based follow‐up care system for patients with burn in Iran: Participatory action research

**DOI:** 10.1002/nop2.483

**Published:** 2020-04-13

**Authors:** Mojgan Lotfi, Vahid Zamanzadeh, Ali Ostadi, Maryam Jalili Fazel, Afsaneh Nobakht, Mohammad Khajehgoodari

**Affiliations:** ^1^ Department of Medical Surgical Nursing Faculty of Nursing and Midwifery Sina Hospital Tabriz University of Medical Sciences Tabriz Iran; ^2^ Department of Medical Surgical Nursing Faculty of Nursing and Midwifery Tabriz University of Medical Sciences Tabriz Iran; ^3^ Department of Internal Medicine Faculty of Medicine Sina Hospital Tabriz University of Medical Sciences Tabriz Iran; ^4^ Sina Hospital Research Center Tabriz University of Medical Sciences Tabriz Iran; ^5^ Faculty of Nursing and Midwifery Sina Hospital Tabriz University of Medical Sciences Tabriz Iran

**Keywords:** burn, follow‐up care, nurses, nursing, participatory action research

## Abstract

**Aim:**

After the discharge of patients with burns, quality of life, psychological and social adjustment, performance and their follow‐up are ambiguous. Therefore, we decided to improve the status of family‐based care programmes in patients with the burn**.**

**Design:**

Participatory action research.

**Methods:**

The participatory action research was conducted between the Faculty of Nursing, Sina Hospital's managers and multidisciplinary burn teams from 2017–2018. The procedure for data collection included focus group meetings with key informants, interviews, observation, and questionnaire. Qualitative data were analysed using the qualitative content analysis and qualitative data were analysed by SPSSv.24.

**Results:**

The study, comprised of four phases, started in May 2017 and completed in 9 months. The results of quantitative showed that quality of life has a statistically significant difference before and after the action. The qualitative data resulted were grouped into 3 categories and 28 subcategories and were analysed in the SWOT Matrix. All the multidisciplinary burn teams together with the managers as a team working of the care providers and the academic researcher resulted in enablers the changes in providing health education and services as well as improving the quality of life of patients and their families.

## 1 INTRODUCTION

All around the world, burn injuries are one of the most devastating forms of trauma (Outwater, Ismail, Mgalilwa, Justin Temu, & Mbembati, 2013; Rani & Schwacha, 2011) that lead to unpleasant consequences that can have an impact on all aspects of life, such as psychological, social and physical functioning (Bosworth‐Bousfield 2002). It is an injury to the skin, or other organic tissues destroyed some or all of the skin cells by hot liquids, hot solids, flames, radiation, radioactivity, chemicals, friction and electricity (Abu‐Sittah, Chahine, & Janom, 2016; Peck, 2011).

## 2 BACKGROUND

Annually, more than 300,000 people die as a result of burn injuries, in which 95% occur in low‐ and middle‐income countries (Tyack, Simons, Spinks, & Wasiak, 2012). In Iran, 150,000 burns occur each year, with an annual death of 3,000 individuals (Saberi, Fatemi, Soroush, Masoumi, & Niazi, 2016).

However, nowadays with the development of specialized burn centres and associated advances in treatment, mortality rates of these patients have been decreased, but burns still lead to complex metabolic changes that can adversely affect the whole body system (Brusselaers, Monstrey, Vogelaers, Hoste, & Blot, 2010; Herndon, 2018).

Scarring, deformity and dysfunctions are challenges of postdischarge and lead to a delay in the recovery process. Patients often experience an abnormal appearance even after receiving the most advanced treatment. Life with the scars can be problematic and has a major impact on the individual's life, especially in social–cultural situations, which costs a lot for physical attractiveness (Guest, Griffiths, Harcourt, & healing, 2018), and it has a huge economic burden on patients, their families, insurance companies and healthcare systems (Seyed‐Forootan et al., 2016).

Physical, mental and educational performance of most patients after burns has decreased, and, as a result, frequent absence from work, school and university is observed in these patients.

Therefore, discharge from the hospital does not mean the end of treatment for a patient with burns, but beginning of a new period of care. In fact, the care responsibility of the treatment team is transferred to the patient and family which may be associated with new problems (Coleman et al., 2004).

Given the psychological and functional changes that result from burns, discharging from the hospital is often scary for the patient with burn and his/her family. And patients should be coping with these changes without the presence of care team especially nursing care (Barnason, Zimmerman, Nieveen, & Hertzog, 2006). After discharge, nearly 19% of patients with burns experience incompatible events (Forster, Murff, Peterson, Gandhi, & Bates, 2003). Therefore, the need for care and rehabilitation such as mental health, attention to burned skin and wound care, physiotherapy, nutrition, pain management, temperature regulation, resumption of sexual relations, follow‐up treatment and surgical repair, economic problems, return to the workplace and the presence without fear and embarrassment in the community is necessary after hospital discharge for these patients (Hinkle & Cheever 2018). For these reasons, follow‐up and the importance of educating are important for the patient and his/her family in self‐care (Asgari et al., 2016). Follow‐up care is a regular process for effective communication and interaction between patient and nurse (Molazem, Rezaei, Mohebbi, Ostovan, & Keshavarzi, 2013) and one of the most important ways to help patients with burns in preventing further complications (Holavanahalli, Helm, & Kowalske, 2016). Implementing this programme is more effective if it comes with the close involvement of the family and the follow‐up of the nursing system (Frivold, Slettebø, Heyland, & Dale, 2018). Therefore, there is a need for a coherent and principled programme for discharge and designing follow‐up care programmes for these patients (Jafaryparvar, Adib, Ghanbari Khanghah, & Kazem Nezhad Leyli, 2018).

Considering the importance of rehabilitation in these patients, the lack of a follow‐up care programme and the existing study gap in the field of discharge and follow‐up programmes for these patients in Iran, we decided to use the action research method to improve the status of family‐based care programmes for these patients. Therefore, this study was designed to develop a family‐based follow‐up system for patients with the burn.

## 3 METHODS

This study is based on a participatory action research (PAR) approach to the development of family‐based follow‐up care system for patients with burn who are discharged from hospital and require nursing care. The study was approved by the institutional review board of Tabriz University of Medical Sciences in Iran.

Action research combines essential practical work with the research process, and it is an action which the research organizes it. It is an effort on the way to cognitive which is achieved by addressing the process of improvement and recovery (Danley & Ellison, 1999; Elliot, 1993). Action research by pursuing its goal (problem‐solving and reform) has more emphasis on representation, performance and the possibility of considering the issue (Zeichner, 2008).

In action research, the researcher is not the sole conductor of the research, but the participants are members of the research group (Danley & Ellison, 1999). The PAR is a qualitative methodology that involves asking stakeholders who have a specific interest in the use of the research product to answer the research questions (Campbell & Murray, 2004; Casey, 2007).

Considering that the aim of this study is to improve follow‐up programmes after the discharge of burn patients based on family‐based care and the achievement of this goal is based on the coherence of teamwork of various professional groups and in close connection with the existing system, therefore, we will use the participatory action research methodology in this research. We have followed Reason and Bradbury (2001) model, which included four stages in the PAR (planning, action, observing and reflection) that can be repeated in cycles until we achieve our objectives (Reason & Bradbury, 2001).

### 3.1 Setting

This study was conducted at Sina Tabriz hospital which is known as the Northwest Burns Referral Center of Iran. Sina hospital with 78 active beds for patients (the women's burn ward has 19 beds, the men's and paediatric burn ward each has 18 beds, reconstruction wards has 14 beds, and BICU has 9 beds), clinic of burn and physiotherapy admits approximately 1,200 burned patients annually. The families speak Persian, Azerbaijani and Turkish.

### 3.2 Participants

A total of 14 primary co‐researchers including four nursing supervisors, nursing manager, three nurses, two head nurses, head of the hospital and three faculty members formed the inquiry group.

Secondary participants include other multidisciplinary burn teams such as a burn specialist, a pharmacist, a dietitian, a pulmonary specialist, a psychologist, a social doctor, an infectious specialist and a physiotherapist.

Totally, there were 11 women and 11 men with a mean age of 33 years (range = 23–47) and average work experience of eight years (range = 5–15). In addition, a consecutive sampling method was used through which 70 patients and their records were assessed in follow‐up care system over a 6‐month period from August 2017–February 2018.

### 3.3 Procedures

The first cycle of the study, comprised of four phases, started in May 2017 and completed after 9 months.

#### 3.3.1 Phase 1 (Initial reflecting & planning)

The first phase of the study participatory action began with data collected by the research team with an extensive study. Qualitative data were collected in the form of group discussion with participants, among 8 focus group (F.G) meetings (once a week).

In the initial F.G discussion, a semi‐structured questionnaire was used to elicit participant stories of ideal and current practice. Gaps and opportunities were discussed by participants. All F.G lasted 60–90 min and were facilitated by the first authors using a question guide. After recognizing the need for change, the solutions were assessed with more details.

Then, an action plan was formulated with the objective identifying the educational needs of patients who are being discharged and follow‐up that included the activities, people, resources and timelines to accomplish each task. The importance of identifying the level of patients' satisfaction with the educational performance, wound assessment and follow‐up of the nurses during the study accepted by the group.

#### 3.3.2 Phase 2 (Act)

In the second phase, the participants were engaging in actions based on the action plan.

The head nurses in the burn wards introduced the discharge patients to the educator nurse. Patient training needs were identified by the educator nurse, and the patient's record was completed by the educator in the ward; then, care objectives agreed with the patient and one of the relatives.

The time of referrals and subsequent follow‐up was documented in the record. A pamphlet, booklet and card contained the profile of the care and follow‐up file after the discharge, and the phone number was delivered to the patient and family for necessary contacts. It was emphasized in case of any problem or question, he/she could contact the number entered on the card. The name, telephone number or mobile number and patient address were registered in the follow‐up file. Family‐based follow‐up care was organized according to the chart, and with the presence of the patient and a relative at the hospital, the nursing care and counselling required was carried out by the co‐researchers. In the absence of a patient referral, the follow‐up and consultation were provided by the co‐researchers. The patient was referred to the burn specialist or other medical professionals if needed common topical or systemic therapies interventions such as prescribing creams and ointments, medications, sedation, anti‐itching, vitamins and dietary supplements. Additionally, patients were advised to visit nutrition counsellors and physiotherapists if necessary. Finally, all the information was registered in the patient's record.

#### 3.3.3 Phase 3 (Observe)

Head nurses, clinical and educational supervisors, and a nursing faculty member regularly monitor discharged patients, their educational needs, documentation, medical and nursing care and patient follow‐up.

In this phase, the strengths and weaknesses of the study conducted in previous cycle and corrective actions needed for the next cycle in the focus group were discussed.

Patients' satisfaction with advisory services and follow‐up after discharge programme, independent performance, quality of life, documentation (related to the follow‐up) by face to face or by telephone, surveys from consulting and follow‐up team members and survey of the officials and project managers using separate checklist were evaluated and analysed.

#### 3.3.4 Phase 4 (Reflection)

**FIGURE 1:**
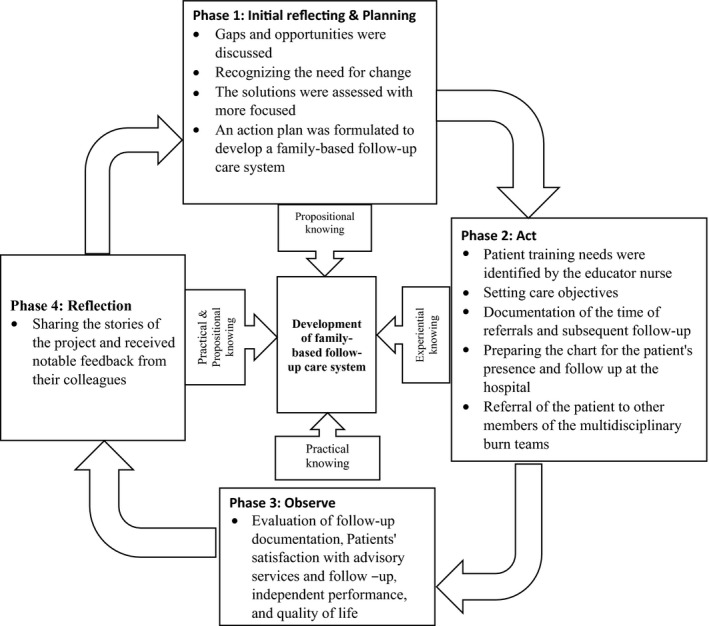
Schematic diagram of the phases to develop a family‐based follow‐up care system for patients with burn in Iran

During reflection meeting, participants shared their story, reflections, observations and feeling of the project and received notable feedback from their colleagues. However, the reflection also took place in the middle of ongoing action in informal conversations and consultations between the participants. The F.G did not continue until consensus occurred on each domain (Figure 1).

### 3.4 Ethical consideration

Approval the project was obtained from the university ethics boards with number TBZMED.REC.1394.462. The permission of the hospital management and nurse manager was obtained before the study. The data were managed using ethical principles. All information that was obtained through observation, interviewing, meeting and recording from the meeting, patients and the relatives were kept confidential and names and identifying information removed.

### 3.5 Data analysis

Data in action research can be organized based on participant stories, interpretation and action (Williamson, Bellman, & Webster, 2011). Qualitative content analysis was used to reveal hidden semantic patterns of qualitative information. The facilitator listened carefully to the audio data collected at the group discussion, verified, coded and categorized transcript data. Constant comparison technique and an inductive approach were performed to translate the results of the reflection meetings to propositional knowledge (Polit & Beck, 2012).

First, data were organized by the interview questions and, second, organized under the SWOT (Strengths, Weakness, Opportunities and Threats). The PAR findings of both the participants' perception should be correlated and increase their awareness of reality (Coughlan & Coghlan, 2002).

Quantitative data by means the Persian version of Burn Specific Health Scale‐Brief Questionnaire (BSHSB) were carefully examined to assess the quality of life which consists of 40 items expressing concern skin sensitivity to heat, body image, hand performance, how to take care of burned areas, occupation–communication and ability to perform simple activities, sexual performance and emotional aspect. This questionnaire consists of 40 items. From which, items 18, 11 and 12 are related to the physical, psychological and social aspects of quality of life, respectively. The 5‐point Likert items included “very high,” “somewhat high,” “moderate,” “low” and “in no way.” The score ranges from 1–5, respectively. Quality of life is determined by the total scores of the responses to the questionnaire. Reliability of questionnaire was confirmed by a test–retest method with a correlation coefficient of 0.85. The checklist was designed to assess the educational needs of patients, training provided, patient's referral times, the reason for referral and actions taken at the time of referral to the patient.

### 3.6 Rigour

Considering the similarities of the qualitative researches challenges, in this study we have also used the rigorous criteria pertaining to qualitative research including dependability, credibility, transferability and confirmability (Lincoln & Guba, 1994). Semi‐structured interviews in the meeting, field notes, reflections, questionnaire and checklist contributed to method triangulation and comply with study dependability. Credibility was promoted by the first author's immersion in the subject matter and sent where the preliminary results to the participants to assess their initial statements and the identified codes. Transferability was demonstrated using descriptions with direct quotations. Two colleagues with experience in qualitative research review all data and documents to verify confirmability.

## 4 RESULTS

### 4.1 Quantitative

Analysis of BSHSB Questionnaire scoring was made using SPSS software version 24. A total of 70 patient participated in the quality of life ranking, and all participants before and 64 participants after establishing the family‐based follow‐up care system completed the questionnaire.

Education level in most of the participants was elementary, with an average age of 39.45 and the mean burned of total body surface area (TBSA) of 14.63%. The normal distribution of data was confirmed using the Kolmogorov–Smirnov test. The comparison of quality of life before and after the family‐based follow‐up care system was performed using the paired *t* test.

The results showed that quality of life has a statistically significant difference. The mean level of quality of life score increased to 175.29 after implementing interventions in contrast to 156.76 before it (Table 1). Multiple linear regression was calculated to predict the quality of life scores based on their burn percentage, age, stay in the hospital and burn per cent. It was found that patients' quality of life is reduced by 0.898 units for each burn percentage.

**TABLE 1:** Comparison of patients/families quality of life scores of patients before and after implementing family‐based follow‐up care system in the burn wards

Group	Mean & *SD*	*p* value
Before action	143.90 ± 2.75	.001
After action	175.29 ± 2.68	

### 4.2 Qualitative

Through the reflection phase, the participants concluded that patients and their families, after discharge from the hospital, need to receive numerous health information and support. Assessment and analysing the qualitative data resulted in findings, and 28 subcategories related to the development of family‐based follow‐up care system for patients with burn in Iran. These findings were grouped into 3 categories (obstacles encountered, enablers encountered and strategies and actions). All 28 subcategories are included in the SWOT matrix (Table 2). Subsequently, some of the obstacles and enablers which were targeted for intervention are described here by verbatim quotes from the focus group transcriptions.

**TABLE 2:** A strategic planning technique used to identify the strengths, weakness, opportunities and threats to develop a family‐based follow‐up care system

Internal factors	Strengths (S)		Weakness (s)
External factors	1. The support of the programme by nursing and hospital managers 2. Learning of training practice 3. Giving specific training 4. Supervising supervisor and head nurses 5. Helping in making change 6. Doctors support the programme 7. Create a responsive system to respond to the needs of patients and their families	8. Reduce the waiting time for patients to receive care and follow‐up 9. Possibility to create patient education channel 10. Qualified personnel for specialized nursing care in the burn 11. The existence of patterns and models based on context theories	The difficulty of communication with patients in distant areas Lack of specific instructions for laws, regulations and legal issues The absence of specific instructions to patient tariffs for nursing services

Opportunities (O)		SO Strategy	WO Strategy
	Patients and families welcome the method of work Supporting the Ministry of Health's Nursing Assistant Improving patient and families’ communication by the hospital Improving physical and mental activities Increased satisfaction of patients and families Reduced re‐hospitalization Creating social networks for patient interaction Having specific instructions for home care Use of master's degree students and Ph.D. students nursing to participate in the programme Use of individuals with qualifications and licensed to provide care at home	B + 1, 4, 5, 6 J + 2, 3, 10 A, C, D, E, G + 5, 6, 7, 8 B, E, F, I + 1, 7, 9, 10, 11	B, G, H, I, J + 1 A, B, H + 3 A, B, E, H + 4

Threats(T)		ST Strategy	WT Strategy
	Personnel shortage Lack of equipment Failure to implement a home care programme by the hospital	A, B + 3, 7, 8, 9, 10 C + 3, 10, 11	A + 1, 3, 4 B + 2, 4 C + 1, 2, 4

### 4.3 Shortage of resources

Participants believed that to continue the programme within 24 hr, the continuing presence of the treatment team is required. “We are doing this now, but for the coming years, we need to consider the required expert team” (Int‐G). There is no required equipment such as a computer system, a telephone, a proper documentation system and cars for transferring the treatment team to visit patients at home. Also, a lack of financial resources and the non‐existence of a budget for continued training were also identified as added threats to this study.

### 4.4 Low access to health services

The participants claim that visiting patients in remote or rural areas is difficult: “…because we do not have the necessary facilities to go to these areas and patients can not afford to come to the center for care and treatment” (Int‐G). It was recognized as one of the main threats in this study.

### 4.5 Failure to implement a home care programme by the hospital

None of the country's hospitals have home care programmes for patients who are discharged from the hospital. “…The care provided to patients at home is done by people who do not have qulification and patients with more complex problems are re‐hospitalized for treatment and care…”(Int‐G).

### 4.6 Lack of executive Statute

Another threat encounter by the participants in the group discussions was legal issues in the implementation of the programme: “The participants claim that medical tariffs are known to treat patients, but for nursing care, tariffs are not specified. The legal issues that may arise when taking care or treating patients at home are another challenge encountered by participants…” (Int‐G). In general, there is no comprehensive instruction to all the requirements for the development of family‐based follow‐up care system in the country.

### 4.7 Lack of a specific training for patients at the time of discharge

During the group discussions, it became clear that there was no specific training package for burn patients at the time of discharge and each nurse teaches information to patients based on their knowledge of burn and care: “At the time of the discharge of the patient from the hospital with a personal desire, no training was provided to the patient and family” (Int‐G).

### 4.8 The support of multidisciplinary burn teams to the change

Participants were very happy to be able to help in improving patients' performance for return to a better life. Hospital and nursing managers were seeking solutions to reduce problems. They provided the required facilities as much as possible. Hospital physicians welcomed the programme, and for the better execution of the programme, they offered a variety of suggestions.

The nursing assistant at the Ministry of Health inaugurated the family‐based follow‐up care clinic at the hospital which was characterized by focused group discussions. In general, all people in the multidisciplinary burn teams played an active role in this programme.

### 4.9 Use the capacity of nursing students

The use of master degree nursing students and Ph.D. students of nursing in this programme provides good opportunities for hospitals, patients and students. The nursing manager, with the least possible cost, eliminates the shortage of personnel needed to carry out family‐based follow‐up care. Students, independently or along with other caregivers, take care of their patients at home or at the clinic, and patients are satisfied that those who are constantly following their situation. Therefore, their satisfaction increases with the hospital's services:

Nursing students will also have a good experience in this field and will gain a successful resume. Finally, Students can present their ideas and experiences as quantitative and qualitative research projects. (Int‐Nurse)

### 4.10 Improve patient satisfaction

In this plan, patients were followed up every day. Physical status, nutrition, comfort and psychosocial status were assessed face to face or by telephone. Burn sites were examined separately for repair, infection, appearance, discharge and function. If needed, the patient was referred to special counselling or admission to the hospital immediately: “…The patient's quality of life and performance were assessed monthly” (Int‐Nurse). These steps improved patient satisfaction from hospital services, especially nursing care.

### 4.11 Strategies and actions

The strategies and actions applied to the development of family‐based follow‐up care system for use and guidance of hospitals are discussed below:

#### 4.11.1 Formation of research team

With the efforts of researchers who have the clinical experience of burns formed four meeting with a team of hospital managers, nursing managers and nurses to investigate the problems after the discharge of patients with burns in May 2017. All participants agreed to achieve positive outcomes to improve the level of care provided to patients during and after hospitalization. One of the outcomes of the meeting with the primary participants was that the multidisciplinary burn teams participated in the project.

#### 4.11.2 Involvement of multidisciplinary burn teams

In June 2017, a meeting was held with the primary participation of participants (managers and nurses) and secondary participants (multidisciplinary burn teams) for a better study during hospitalization and after the discharge of patients with burns. The most important issues discussed by the participants include determining the training packages during hospitalization and after the discharge by multidisciplinary burn team and identify one or more person interested in teaching to educate patients and engaging patients in their care and treatment at period hospitalization.

#### 4.11.3 Determine specific training for burn patients

The burn training programme was designed in the practical form with the participation of multidisciplinary burn team, and this training programme was taught by the educator nurse to the patients. Prior to the project, the caring and therapeutic training had a theoretical aspect and more for nurses and other caregivers than for specialized training of burn patients. The educator nurse believes that: “…I now makes the necessary training technique in a simple and understandable way for patients and they are very satisfied with this teaching method” (Int‐Nurse).

#### 4.11.4 Launching room for examination, follow‐up and documentation patients information

When the project was done actively and special training was given to patients, the first follow‐up was announced to patients by giving them a phone number but there was not a proper place to check patients at the time of the first follow‐up and other follow‐ups:

Patients referred to several places such as burn wards, the office of nurse managers and the doctors' room to be examined. We should take with ourselves things such as dressing, sterile gloves, washing serum, etc, plus a room to examine any patient who came to follow up and examination. (Int‐Nurse)

A meeting was held with participation of research participants due to a large number of patients welcomed this project and the lack of a separate unit with appropriate facilities such as dressing, telephone, computer, and bed or stretcher. It was notable, however, in F.G discussion approved a room called the Nursing Care Room delivered to participants to assess patients.

## 5 DISCUSSION

Identifying patients who have been burned, their physical and mental status, their place of residence and their economic status and family support will help the burn treatment team to provide the necessary conditions for improving patients' quality of life through regular follow‐up.

The level of performance of patients at home and at work, their quality of life and wound healing after discharge should be followed up by hospital officials. Unfortunately, there is no follow‐up care system not only for patients with burns but also for other patients after discharge in Iran (Lotfi, Zamanzadeh, Valizadeh, &Khajehgoodari, 2019).

Once a member stated: “Some families were hospitalized in this center for burning due to a gas explosion. They had a high burn percentage and their home were completely burned. Our concern after their discharge was where they were going to live. They had no home. Who should take care of their burn wounds?” (Int‐Nurse).

Launching and implementing follow‐up care reduce hospitalization costs and reduce the workload of personnel, as well as improving the quality of life of patients. One participant stated: “Many patients prefer to be discharged sooner from hospital due to hospital costs and little attention is paid to the care required for burns. After a period of time, due to lack of wound healing, wound infections and/or changes in the function of burned organs are re‐hospitalized… but this follow up has been a great help for these patients”. Another participant stated: “The rate of burn complications and re‐admission of these patients has decreased because they are continually being followed‐up...”

The study of Heydarikhayat et al, entitled “Effect of post‐hospital discharge follow‐up on health status in patients with burn injuries: a randomized clinical trial,” stated that although burn patients required continued care for pain, psychological health and itching problems, psychological status and scar management were improved due to postdischarge follow‐up health (Heydarikhayat, Ashktorab, Rohani, & Zayeri, 2018). The participants could learn how to collectively change their practice through a systematic process of recognizing barriers against intended action, formulating the action plan, engaging in action and evaluating the impacts of innovations on their patient’ satisfaction and quality of life.

In most of the focused group discussions, there was a consensus that all members of the burn treatment team (burn specialist, nurse, pharmacist, dietitian, pulmonary specialist, psychologist, social doctor, infectious specialist and physiotherapist) should be active and involved in follow‐up care. One participant mentioned: “The team should provide continuous counselling and follow up for these patients as long as the patients and families feel they do not need to take care” (Int‐G).

In this study, we evaluated the impact of practice change on patient quality of life using the BSHSB Questionnaire and examination of burn site to give feedback to the inquiry group and to arrange actions based on these measurements. An improvement in the quality of life of patients indicated that the new practices met the practical needs and expectations of the patients and their families. Results of a study by Hashemi et al also showed an increase in the quality of life of burn patients in the time interval of less than two months after the interventions using the Orem Self‐Care Program (Hashemi et al., 2014). In line with other studies, these results showed that the three domains of BSHSB improved after the intervention (Ardebili et al., 2017; Heydarikhayat et al., 2018; Tang et al., 2015).

The presented results are consistent with the study conducted by Radwan et al. entitled “Effect of a rehabilitation programme on the knowledge, physical and psychosocial functions of patients with burns” that indicated implementing a 7‐day rehabilitation programme for 2 weeks would improve the physical, social and mental function of the patients (Radwan, Samir, Aty, & Attia, 2011).

In this study, the process of establishing a family‐based follow‐up care system for patients with burn became actionable through consummating knowledge grounded on the action in the relevant context by the care providers who produced it. Our knowledge is grounded in our experience (experiential knowing), expressed through our stories and images (presentational knowing), understood through ideas which make sense to us (propositional knowing) and expressed in worthwhile activities in our lives (practical knowing) (Reason & Bradbury, 2001).

## 6 CONCLUSION

Co‐operative working of the care providers and the academic researchers resulted in enablers to make basic changes in providing health education and services as well as improving the quality of life of patients and their families. In this study, all the multidisciplinary burn teams together with the managers as a team worked to improve patients' performance and improve their quality of life. This experience offered suggestions that may be helpful to other settings as the professionals wish to move towards innovative practice in the field of health education.

## 7 RELEVANCE TO CLINICAL PRACTICE

Incidence of physical and psychosocial problems due to burn will affect the quality of life of these patients, and usually, the quality of life in burn patients is decreased. In our study, with continuous patient follow‐up, we tried to identify physical and psychological problems with regular examinations and immediate action was taken for necessary advice such as nutrition, psychology and infectiousness.

## CONFLICT OF INTEREST

The authors have no conflicts of interest, including specific financial interests and relationships and affiliations relating to the subject or materials discussed in the manuscript.

## Supporting information

File S1

## References

[nop2483-bib-0001] Abu‐Sittah, G. S. , Chahine, F. M. , & Janom, H. (2016). Management of burns in the elderly. Annals of Burns and Fire Disasters, 29(4), 1–43. PMC5347309 28289356

[nop2483-bib-0002] Ardebili, F. M. , Ghezeljeh, T. N. , Bozorgnejad, M. , Zarei, M. , Ghorbani, H. , & Manafi, F. (2017). Effect of multimedia self‐care education on quality of life in burn patients. World Journal of Plastic Surgery, 6(3), 292. 29218277 PMC5714973

[nop2483-bib-0003] Asgari, P. , Mahmoudi, M. , Hekmatpou, D. , KhajehGoodari, M. , Rafiei, F. , & Tajik, R. (2016). The effect of education of occupational safety on knowledge and improvement of employee performance during moving patients in intensive care units. Iran Occupational Health, 13(5), 71–79.

[nop2483-bib-0004] Barnason, S. , Zimmerman, L. , Nieveen, J. , & Hertzog, M. (2006). Impact of a telehealth intervention to augment home health care on functional and recovery outcomes of elderly patients undergoing coronary artery bypass grafting. Heart and Lung, 35(4), 225–233. 10.1016/j.hrtlng.2005.10.003 16863894

[nop2483-bib-0005] Bosworth‐Bousfield, C. (2002). Burn trauma management and nursing care (2nd ed.). London, UK: Whirr Publishers.

[nop2483-bib-0006] Brusselaers, N. , Monstrey, S. , Vogelaers, D. , Hoste, E. , & Blot, S. (2010). Severe burn injury in Europe: A systematic review of the incidence, etiology, morbidity and mortality. Critical Care, 14(5), R188 10.1186/cc9300 20958968 PMC3219295

[nop2483-bib-0007] Campbell, C. , & Murray, M. (2004). Community Health Psychology: Promoting analysis and action for social change. Journal of Health Psychology, 9(2), 187–195. 10.1177/1359105304040886 15018722

[nop2483-bib-0008] Casey, D. (2007). Using action research to change health‐promoting practice. Nursing & Health Sciences, 9(1), 5–13. 10.1111/j.1442-2018.2007.00297.x 17300539

[nop2483-bib-0009] Coleman, E. A. , Smith, J. D. , Frank, J. C. , Min, S. J. , Parry, C. , & Kramer, A. M. (2004). Preparing patients and caregivers to participate in care delivered across settings: The care transitions intervention. Journal of the American Geriatrics Society, 52(11), 1817–1825. 10.1111/j.1532-5415.2004.52504.x 15507057

[nop2483-bib-0010] Coughlan, P. , & Coghlan, D. (2002). Action research for operations management. International Journal of Operations & Production Management, 22(2), 220–240.

[nop2483-bib-0011] Danley, K. S. , & Ellison, M. L. (1999). A handbook for participatory action researchers. Boston, MA: Boston University Center for Psychiatric Rehabilitation.

[nop2483-bib-0012] Elliot, J. (1993). Action research for educational change. Bulletin of Science, Technology & Society, 13(1), 47–47. 10.1177/027046769301300149

[nop2483-bib-0013] Forster, A. J. , Murff, H. J. , Peterson, J. F. , Gandhi, T. K. , & Bates, D. W. (2003). The incidence and severity of adverse events affecting patients after discharge from the hospital. Annals of Internal Medicine, 138(3), 161–167. 12558354 10.7326/0003-4819-138-3-200302040-00007

[nop2483-bib-0014] Frivold, G. , Slettebø, Å. , Heyland, D. K. , & Dale, B. (2018). Family members' satisfaction with care and decision‐making in intensive care units and post‐stay follow‐up needs—a cross‐sectional survey study. Nursing Open, 5(1), 6–14. 10.1002/nop2.97 29344389 PMC5762765

[nop2483-bib-0015] Guest, E. , Griffiths, C. , & Harcourt, D. (2018). A qualitative exploration of psychosocial specialists' experiences of providing support in UK burn care services. Scars, Burns & Healing, 4, 2059513118764881 10.1177/2059513118764881 PMC5987094 29873339

[nop2483-bib-0016] Hashemi, F. , Dolatabad, F. R. , Yektatalab, S. , Ayaz, M. , Zare, N. , & Mansouri, P. (2014). Effect of Orem self‐care program on the life quality of burn patients referred to Ghotb‐al‐Din‐e‐Shirazi burn center, Shiraz, Iran: A randomized controlled trial. International Journal of Community Based Nursing and Midwifery, 2(1), 40. 25349844 PMC4201185

[nop2483-bib-0017] Herndon, D. N. (2018). Total burn care. London, UK: Saunders.

[nop2483-bib-0018] Heydarikhayat, N. , Ashktorab, T. , Rohani, C. , & Zayeri, F. (2018). Effect of post‐hospital discharge follow‐up on health status in patients with burn injuries: A randomized clinical trial. International Journal of Community Based Nursing and Midwifery, 6(4), 293. 30465002 PMC6226610

[nop2483-bib-0019] Hinkle, J. L. , & Cheever, K. H. (2013). Study Guide for Brunner & Suddarth's Textbook of Medical‐ surgical nursing. Philadelphia, PA: Lippincot Williams and Wilkins.

[nop2483-bib-0020] Holavanahalli, R. K. , Helm, P. A. , & Kowalske, K. J. (2016). Long‐term outcomes in patients surviving large burns. Journal of Burn Care & Research, 37(4), 243–254. 10.1097/BCR.0000000000000257 26056761

[nop2483-bib-0021] Jafaryparvar, Z. , Adib, M. , Ghanbari Khanghah, A. , & Kazem Nezhad Leyli, E. (2018). Quality of life and associated factors in patients suffering from burns. Journal of Holistic Nursing and Midwifery, 28(3), 179–184. 10.29252/hnmj.28.3.179

[nop2483-bib-0022] Lincoln, Y. S. , & Guba, E. G. (1994). Competing paradigms in qualitative research. Handbook of Qualitative Research, 2, 163–194.

[nop2483-bib-0023] Lotfi, M. , Zamanzadeh, V. , Valizadeh, L. , & Khajehgoodari, M. (2019). Assessment of nurse–patient communication and patient satisfaction from nursing care. Nursing open., 6(3), 1189–96. 31367445 10.1002/nop2.316 PMC6650658

[nop2483-bib-0024] Molazem, Z. , Rezaei, S. , Mohebbi, Z. , Ostovan, M.‐A. , & Keshavarzi, S. (2013). Effect of continuous care model on lifestyle of patients with myocardial infarction. ARYA Atherosclerosis, 9(3), 186–191. 23766775 PMC3681277

[nop2483-bib-0025] Outwater, A. H. , Ismail, H. , Mgalilwa, L. , Justin Temu, M. , & Mbembati, N. A. (2013). Burns in Tanzania: Morbidity and mortality, causes and risk factors: A review. International Journal of Burns and Trauma, 3(1), 18–29. 23386982 PMC3560491

[nop2483-bib-0026] Peck, M. D. (2011). Epidemiology of burns throughout the world. Part I: Distribution and risk factors. Burns, 37(7), 1087–1100. 10.1016/j.burns.2011.06.005 21802856

[nop2483-bib-0027] Polit, D. F. , & Beck, C. T. (2012). Nursing research: Generating and assessing evidence for nursing practice. Philadelphia, PA: Wolters Kluwer Health/Lippincott Williams & Wilkins.

[nop2483-bib-0028] Radwan, M. , Samir, S. , Aty, O. , & Attia, S. (2011). Effect of a rehabilitation program on the knowledge, physical and psychosocial functions of patients with burns. Journal of American Science, 7, 427–434.

[nop2483-bib-0029] Rani, M. , & Schwacha, M. G. (2011). Aging and the pathogenic response to burn. Aging and Disease, 3(2), 171–180. 22724078 PMC3377829

[nop2483-bib-0030] Reason, P. , & Bradbury, H. (2001). Handbook of action research: Participative inquiry and practice. Thousand Oaks, CA: Sage.

[nop2483-bib-0031] Saberi, M. , Fatemi, M. , Soroush, M. , Masoumi, M. , & Niazi, M. (2016). Burn epidemiology in Iran: A meta‐analysis study. Iranian Journal of Surgery, 24(1), 47–61.

[nop2483-bib-0032] Seyed‐Forootan, K. , Karimi, H. , Motevalian, S. A. , Momeni, M. , Safari, R. , & Ghadarjani, M. (2016). LA50 in burn injuries. Annals of Burns and Fire Disasters, 29(1), 14–17. 27857645 PMC5108221

[nop2483-bib-0033] Tang, D. , Li‐Tsang, C. W. P. , Au, R. K. C. , Li, K.‐C. , Yi, X.‐F. , Liao, L.‐R. , … Liu, C.‐S. (2015). Functional outcomes of burn patients with or without rehabilitation in mainland China. Hong Kong Journal of Occupational Therapy, 26(1), 15–23.

[nop2483-bib-0034] Tyack, Z. , Simons, M. , Spinks, A. , & Wasiak, J. (2012). A systematic review of the quality of burn scar rating scales for clinical and research use. Burns, 38(1), 6–18. 10.1016/j.burns.2011.09.021 22047828

[nop2483-bib-0035] Williamson, G. R. , Bellman, L. , & Webster, J. (2011). Action research in nursing and healthcare. Thousand Oaks, CA: Sage.

[nop2483-bib-0036] Zeichner, K. M. (2008). A critical analysis of reflection as a goal for teacher education. Educação & Sociedade, 29(103), 535–554. 10.1590/S0101-73302008000200012

