# Supplementary material for: Development of family‐based follow‐up care system for patients with burn in Iran: Participatory action research
Source: Nurs Open. 2020 Apr 13;7(4):1101–9. doi: 10.1002/nop2.483 (PMC7308696; doi:10.1002/nop2.483)
Supplement: Supplementary file 1 — File S1 [file NOP2-7-1101-s001.docx]

Supplementary File 1: Consolidated criteria for reporting qualitative studies (COREQ): 32-item checklist

| No Item | Guide questions/description | Page / response |
| --- | --- | --- |
| **Domain 1: Research team and reflexivity**  Personal Characteristics | | |
| 1. Interviewer/facilitator | Which author/s conducted the interview or focus group? | All authors |
| 2. Credentials | What were the researcher’s credentials? E.g. PhD, MD | Ph. D. and Master of sciences in nursing |
| 3. Occupation | What was their occupation at the time of the study? | Associate Professor of Nursing and practice nurse |
| 4. Gender | Was the researcher male or female? | First author: Female  Correspond author: Male |
| 5. Experience and training | What experience or training did the researcher have? | Burn researcher, wound Nurse, and teacher of burn wounds |
| Relationship with participants | | |
| 6. Relationship established | Was a relationship established prior to study commencement? | Yes, two were members of the hospital's nurse recruitment research team. |
| 7. Participant knowledge of the interviewer | What did the participants know about the researcher? e.g. personal goals, reasons for doing the research | A professional with the aim of promoting nursing care |
| 8. Interviewer characteristics | What characteristics were reported about the interviewer/facilitator? e.g. Bias, assumptions, reasons and interests in the research topic | reasons and interests in the research topic |
| **Domain 2: study design**  Theoretical framework | | |
| Methodological orientation and Theory | What methodological orientation was stated to underpin the study? e.g. grounded theory, discourse analysis, ethnography, phenomenology, content analysis | Participatory Action Research |
| Participant selection | | |
| 10. Sampling | How were participants selected? e.g. purposive, convenience, consecutive, snowball | Purposive and consecutive |
| 11. Method of approach | How were participants approached? e.g. face-to-face, telephone, mail, email | face-to-face  Focus group |
| 12. Sample size | How many participants were in the study? | 22 Participants |
| 13. Non-participation | How many people refused to participate or dropped out? Reasons? | None |
| Setting | | |
| 14. Setting of data collection | Where was the data collected? e.g. home, clinic, workplace | workplace |
| 15. Presence of non-participants | Was anyone else present besides the participants and researchers? | No |
| 16. Description of sample | What are the important characteristics of the sample? e.g. demographic data, date Data collection | Help make a significant change and participating |
| 17. Interview guide | Were questions, prompts, guides provided by the authors? Was it pilot tested? | Participants discussed questions and challenges as appropriate to the issues raised. |
| 18. Repeat interviews | Were repeat interviews carried out? If yes, how many? | yes |
| 19. Audio/visual recording | Did the research use audio or visual recording to collect the data? | yes |
| 20. Field notes | Were field notes made during and/or after the interview or focus group? | yes |
| 21. Duration | What was the duration of the interviews or focus group? | 60–90 minutes |
| 22. Data saturation | Was data saturation discussed? | yes |
| 23. Transcripts returned | Were transcripts returned to participants for comment and/or correction? | yes |
| **Domain 3: analysis and findings**  Data analysis | | |
| 24. Number of data coders | How many data coders coded the data? | 150 |
| 25. Description of the coding tree | Did authors provide a description of the coding tree? | yes |
| 26. Derivation of themes | Were themes identified in advance or derived from the data? | from the data |
| 27. Software | What software, if applicable, was used to manage the data? | SPSS for quantitative data |
| 28. Participant checking | Did participants provide feedback on the findings? Reporting | yes |
| 29. Quotations presented | Were participant quotations presented to illustrate the themes / findings? Was each quotation identified? e.g. participant number | yes |
| 30. Data and findings consistent | Was there consistency between the data presented and the findings? | yes |
| 31. Clarity of major themes | Were major themes clearly presented in the findings? | yes |
| 32. Clarity of minor themes | Is there a description of diverse cases or discussion of minor themes? | yes |
